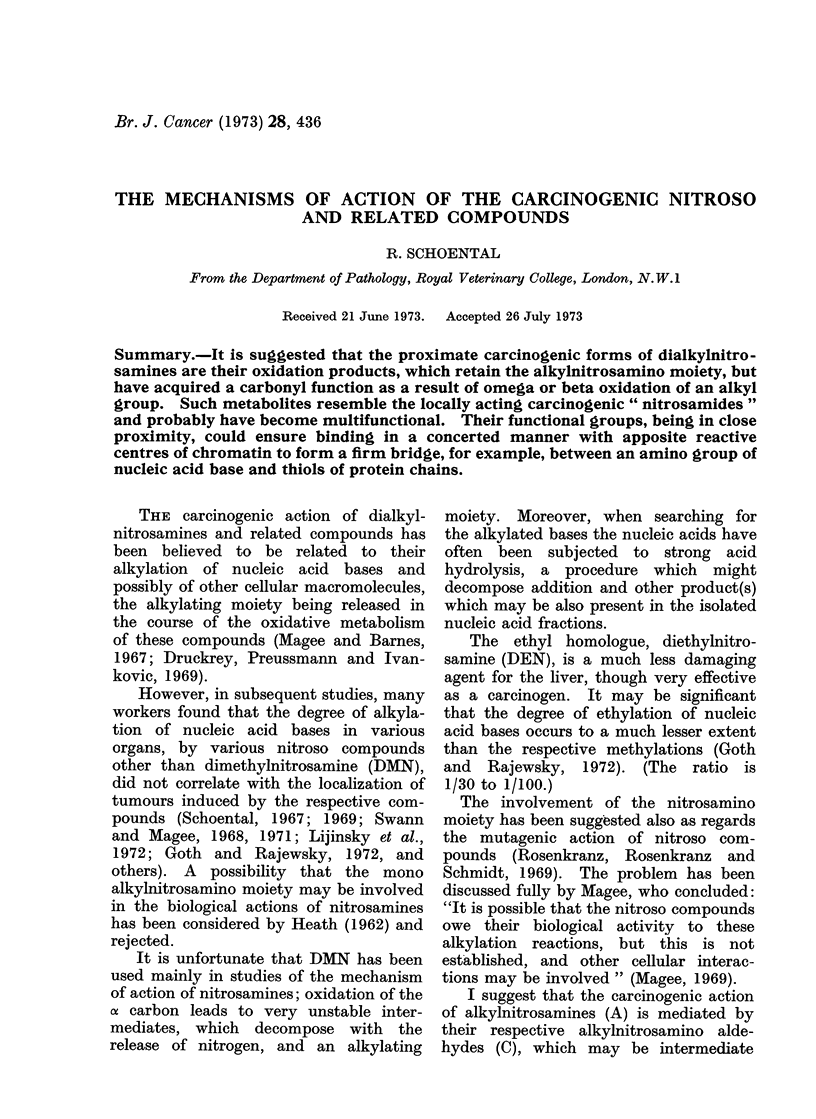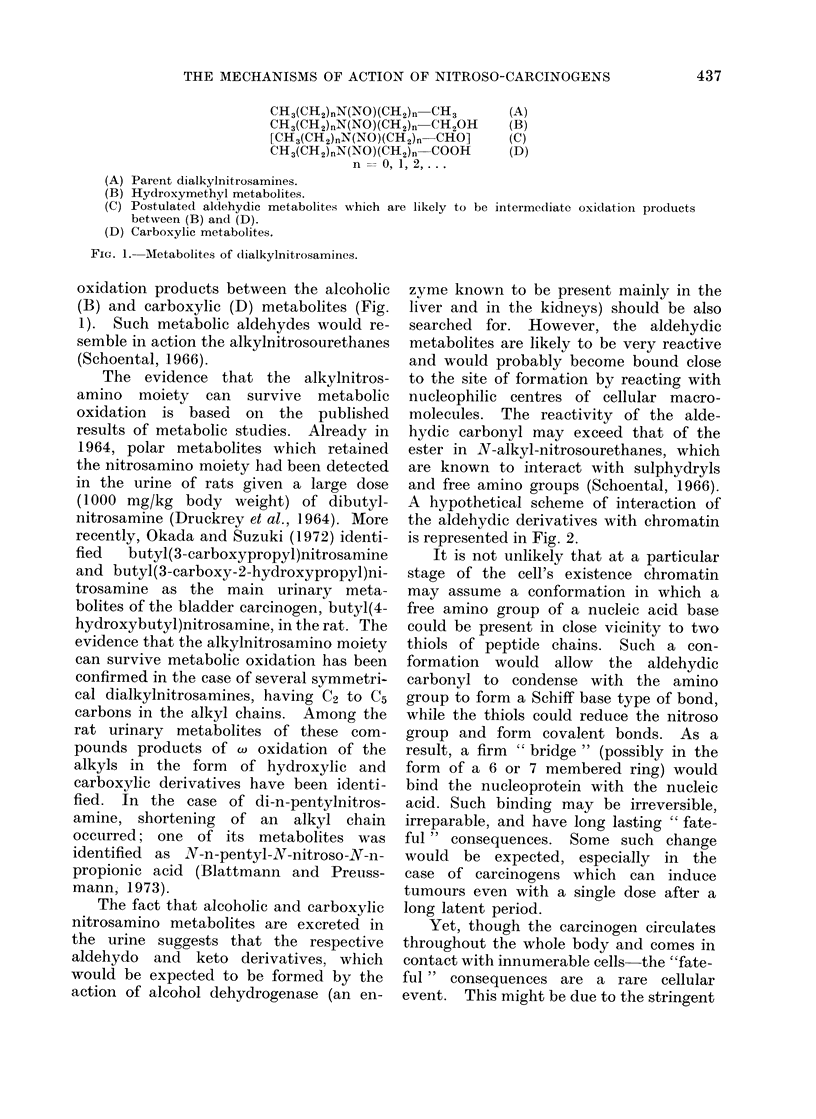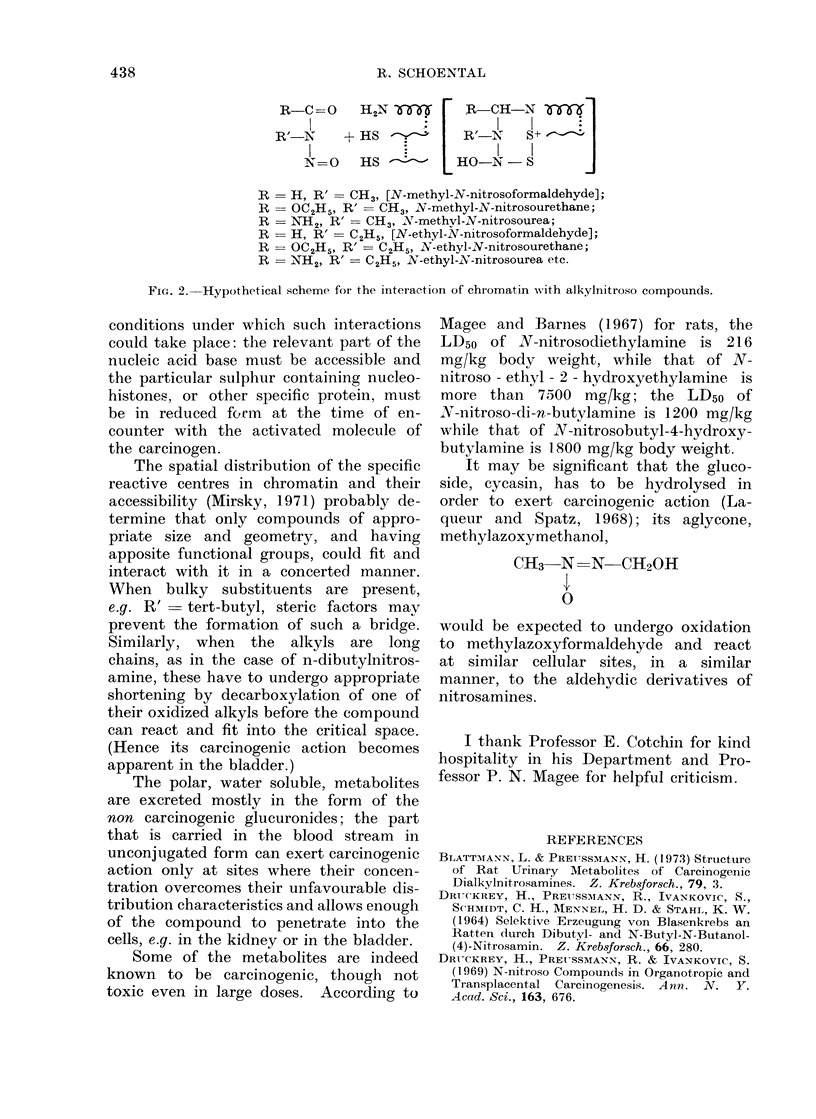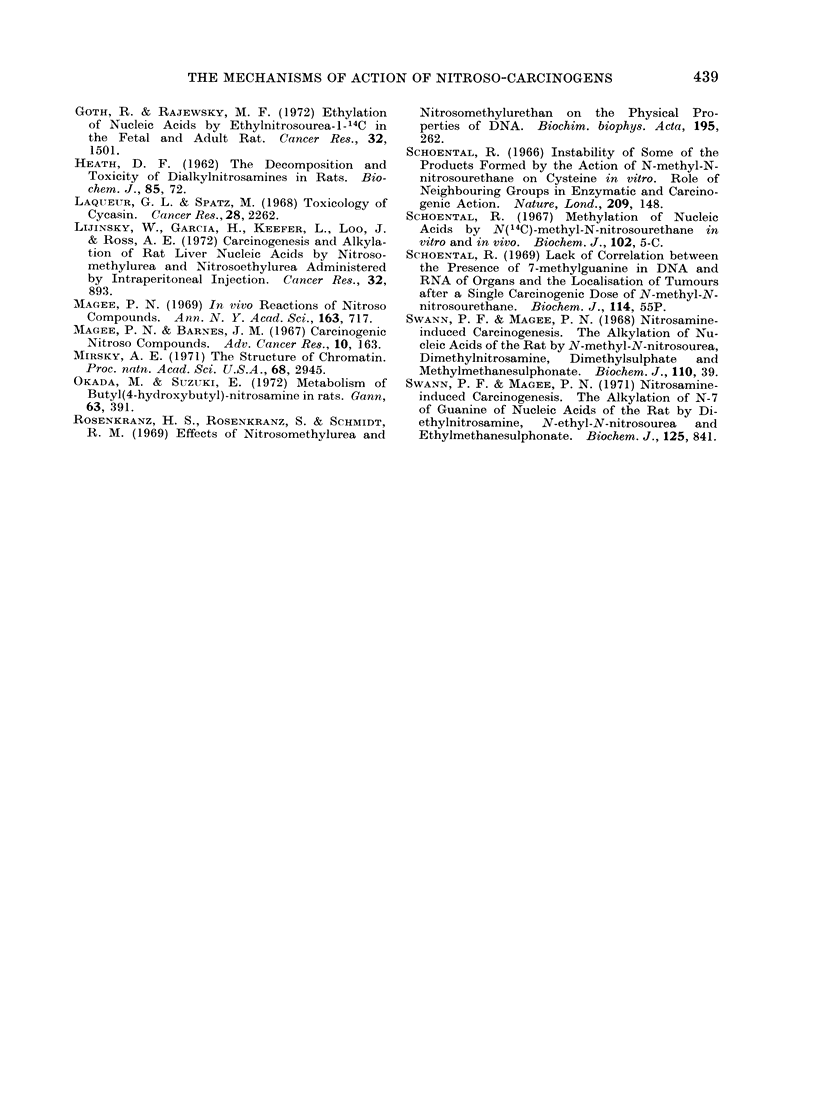# The Mechanisms of Action of the Carcinogenic Nitroso and Related Compounds

**DOI:** 10.1038/bjc.1973.169

**Published:** 1973-11

**Authors:** R. Schoental

## Abstract

It is suggested that the proximate carcinogenic forms of dialkylnitrosamines are their oxidation products, which retain the alkylnitrosamino moiety, but have acquired a carbonyl function as a result of omega or beta oxidation of an alkyl group. Such metabolites resemble the locally acting carcinogenic “nitrosamides” and probably have become multifunctional. Their functional groups, being in close proximity, could ensure binding in a concerted manner with apposite reactive centres of chromatin to form a firm bridge, for example, between an amino group of nucleic acid base and thiols of protein chains.


*Br. J. Cancer* (1973) **28**, 436

### THE MECHANISMS OF ACTION OF THE CARCINOGENIC NITROSO AND RELATED COMPOUNDS

R. SCHOENTAL

*From the Department of Pathology, Royal Veterinary College, London, N.W.1*

Received 21 June 1973. Accepted 26 July 1973

**Summary.—It is suggested that the proximate carcinogenic forms of dialkylnitrosamines are their oxidation products, which retain the alkylnitrosamino moiety, but have acquired a carbonyl function as a result of omega or beta oxidation of an alkyl group. Such metabolites resemble the locally acting carcinogenic " nitrosamides " and probably have become multifunctional. Their functional groups, being in close proximity, could ensure binding in a concerted manner with apposite reactive centres of chromatin to form a firm bridge, for example, between an amino group of nucleic acid base and thiols of protein chains.**

THE carcinogenic action of dialkylnitrosamines and related compounds has been believed to be related to their alkylation of nucleic acid bases and possibly of other cellular macromolecules, the alkylating moiety being released in the course of the oxidative metabolism of these compounds (Magee and Barnes, 1967; Druckrey, Preussmann and Ivankovic, 1969).

However, in subsequent studies, many workers found that the degree of alkylation of nucleic acid bases in various organs, by various nitroso compounds other than dimethylnitrosamine (DMN), did not correlate with the localization of tumours induced by the respective compounds (Schoental, 1967; 1969; Swann and Magee, 1968, 1971; Lijinsky *et al.*, 1972; Goth and Rajewsky, 1972, and others). A possibility that the mono alkylnitrosamino moiety may be involved in the biological actions of nitrosamines has been considered by Heath (1962) and rejected.

It is unfortunate that DMN has been used mainly in studies of the mechanism of action of nitrosamines; oxidation of the $\alpha$ carbon leads to very unstable intermediates, which decompose with the release of nitrogen, and an alkylating moiety. Moreover, when searching for the alkylated bases the nucleic acids have often been subjected to strong acid hydrolysis, a procedure which might decompose addition and other product(s) which may be also present in the isolated nucleic acid fractions.

The ethyl homologue, diethylnitrosamine (DEN), is a much less damaging agent for the liver, though very effective as a carcinogen. It may be significant that the degree of ethylation of nucleic acid bases occurs to a much lesser extent than the respective methylations (Goth and Rajewsky, 1972). (The ratio is 1/30 to 1/100.)

The involvement of the nitrosamino moiety has been suggested also as regards the mutagenic action of nitroso compounds (Rosenkranz, Rosenkranz and Schmidt, 1969). The problem has been discussed fully by Magee, who concluded: "It is possible that the nitroso compounds owe their biological activity to these alkylation reactions, but this is not established, and other cellular interactions may be involved " (Magee, 1969).

I suggest that the carcinogenic action of alkylnitrosamines (A) is mediated by their respective alkylnitrosamino aldehydes (C), which may be intermediate

$$CH_3(CH_2)_nN(NO)(CH_2)_n—CH_3 \quad (A)$$

$$CH_3(CH_2)_nN(NO)(CH_2)_n—CH_2OH \quad (B)$$

$$[CH_3(CH_2)_nN(NO)(CH_2)_n—CHO] \quad (C)$$

$$CH_3(CH_2)_nN(NO)(CH_2)_n—COOH \quad (D)$$

$$n = 0, 1, 2, \ldots$$

(A) Parent dialkylnitrosamines.
(B) Hydroxymethyl metabolites.
(C) Postulated aldehydic metabolites which are likely to be intermediate oxidation products between (B) and (D).
(D) Carboxylic metabolites.

FIG. 1.—Metabolites of dialkylnitrosamines.

oxidation products between the alcoholic (B) and carboxylic (D) metabolites (Fig. 1). Such metabolic aldehydes would resemble in action the alkylnitrosourethanes (Schoental, 1966).

The evidence that the alkylnitrosamino moiety can survive metabolic oxidation is based on the published results of metabolic studies. Already in 1964, polar metabolites which retained the nitrosamino moiety had been detected in the urine of rats given a large dose (1000 mg/kg body weight) of dibutylnitrosamine (Druckrey *et al.*, 1964). More recently, Okada and Suzuki (1972) identified butyl(3-carboxypropyl)nitrosamine and butyl(3-carboxy-2-hydroxypropyl)nitrosamine as the main urinary metabolites of the bladder carcinogen, butyl(4-hydroxybutyl)nitrosamine, in the rat. The evidence that the alkylnitrosamino moiety can survive metabolic oxidation has been confirmed in the case of several symmetrical dialkylnitrosamines, having $C_2$ to $C_5$ carbons in the alkyl chains. Among the rat urinary metabolites of these compounds products of $\omega$ oxidation of the alkyls in the form of hydroxylic and carboxylic derivatives have been identified. In the case of di-n-pentylnitrosamine, shortening of an alkyl chain occurred; one of its metabolites was identified as *N*-n-pentyl-*N*-nitroso-*N*-n-propionic acid (Blattmann and Preussmann, 1973).

The fact that alcoholic and carboxylic nitrosamino metabolites are excreted in the urine suggests that the respective aldehydo and keto derivatives, which would be expected to be formed by the action of alcohol dehydrogenase (an enzyme known to be present mainly in the liver and in the kidneys) should be also searched for. However, the aldehydic metabolites are likely to be very reactive and would probably become bound close to the site of formation by reacting with nucleophilic centres of cellular macromolecules. The reactivity of the aldehydic carbonyl may exceed that of the ester in *N*-alkyl-nitrosourethanes, which are known to interact with sulphydryls and free amino groups (Schoental, 1966). A hypothetical scheme of interaction of the aldehydic derivatives with chromatin is represented in Fig. 2.

It is not unlikely that at a particular stage of the cell's existence chromatin may assume a conformation in which a free amino group of a nucleic acid base could be present in close vicinity to two thiols of peptide chains. Such a conformation would allow the aldehydic carbonyl to condense with the amino group to form a Schiff base type of bond, while the thiols could reduce the nitroso group and form covalent bonds. As a result, a firm " bridge " (possibly in the form of a 6 or 7 membered ring) would bind the nucleoprotein with the nucleic acid. Such binding may be irreversible, irreparable, and have long lasting " fateful " consequences. Some such change would be expected, especially in the case of carcinogens which can induce tumours even with a single dose after a long latent period.

Yet, though the carcinogen circulates throughout the whole body and comes in contact with innumerable cells—the "fateful " consequences are a rare cellular event. This might be due to the stringent

R = H, R′ = $CH_3$, [*N*-methyl-*N*-nitrosoformaldehyde];
R = $OC_2H_5$, R′ = $CH_3$, *N*-methyl-*N*-nitrosourethane;
R = $NH_2$, R′ = $CH_3$, *N*-methyl-*N*-nitrosourea;
R = H, R′ = $C_2H_5$, [*N*-ethyl-*N*-nitrosoformaldehyde];
R = $OC_2H_5$, R′ = $C_2H_5$, *N*-ethyl-*N*-nitrosourethane;
R = $NH_2$, R′ = $C_2H_5$, *N*-ethyl-*N*-nitrosourea etc.

FIG. 2.—Hypothetical scheme for the interaction of chromatin with alkylnitroso compounds.

conditions under which such interactions could take place: the relevant part of the nucleic acid base must be accessible and the particular sulphur containing nucleohistones, or other specific protein, must be in reduced form at the time of encounter with the activated molecule of the carcinogen.

The spatial distribution of the specific reactive centres in chromatin and their accessibility (Mirsky, 1971) probably determine that only compounds of appropriate size and geometry, and having apposite functional groups, could fit and interact with it in a concerted manner. When bulky substituents are present, *e.g.* R′ = tert-butyl, steric factors may prevent the formation of such a bridge. Similarly, when the alkyls are long chains, as in the case of n-dibutylnitrosamine, these have to undergo appropriate shortening by decarboxylation of one of their oxidized alkyls before the compound can react and fit into the critical space. (Hence its carcinogenic action becomes apparent in the bladder.)

The polar, water soluble, metabolites are excreted mostly in the form of the *non* carcinogenic glucuronides; the part that is carried in the blood stream in unconjugated form can exert carcinogenic action only at sites where their concentration overcomes their unfavourable distribution characteristics and allows enough of the compound to penetrate into the cells, *e.g.* in the kidney or in the bladder.

Some of the metabolites are indeed known to be carcinogenic, though not toxic even in large doses. According to Magee and Barnes (1967) for rats, the $LD_{50}$ of *N*-nitrosodiethylamine is 216 mg/kg body weight, while that of *N*-nitroso - ethyl - 2 - hydroxyethylamine is more than 7500 mg/kg; the $LD_{50}$ of *N*-nitroso-di-*n*-butylamine is 1200 mg/kg while that of *N*-nitrosobutyl-4-hydroxybutylamine is 1800 mg/kg body weight.

It may be significant that the glucoside, cycasin, has to be hydrolysed in order to exert carcinogenic action (Laqueur and Spatz, 1968); its aglycone, methylazoxymethanol,

$$\underset{\displaystyle\downarrow\atop\displaystyle O}{CH_3{-}N}{=}N{-}CH_2OH$$

would be expected to undergo oxidation to methylazoxyformaldehyde and react at similar cellular sites, in a similar manner, to the aldehydic derivatives of nitrosamines.

I thank Professor E. Cotchin for kind hospitality in his Department and Professor P. N. Magee for helpful criticism.

### REFERENCES

BLATTMANN, L. & PREUSSMANN, H. (1973) Structure of Rat Urinary Metabolites of Carcinogenic Dialkylnitrosamines. *Z. Krebsforsch.*, **79**, 3.

DRUCKREY, H., PREUSSMANN, R., IVANKOVIC, S., SCHMIDT, C. H., MENNEL, H. D. & STAHL, K. W. (1964) Selektive Erzeugung von Blasenkrebs an Ratten durch Dibutyl- and N-Butyl-N-Butanol-(4)-Nitrosamin. *Z. Krebsforsch.*, **66**, 280.

DRUCKREY, H., PREUSSMANN, R. & IVANKOVIC, S. (1969) N-nitroso Compounds in Organotropic and Transplacental Carcinogenesis. *Ann. N. Y. Acad. Sci.*, **163**, 676.

GOTH, R. & RAJEWSKY, M. F. (1972) Ethylation of Nucleic Acids by Ethylnitrosourea-1-$^{14}$C in the Fetal and Adult Rat. *Cancer Res.*, **32**, 1501.

HEATH, D. F. (1962) The Decomposition and Toxicity of Dialkylnitrosamines in Rats. *Biochem. J.*, **85**, 72.

LAQUEUR, G. L. & SPATZ, M. (1968) Toxicology of Cycasin. *Cancer Res.*, **28**, 2262.

LIJINSKY, W., GARCIA, H., KEEFER, L., LOO, J. & ROSS, A. E. (1972) Carcinogenesis and Alkylation of Rat Liver Nucleic Acids by Nitrosomethylurea and Nitrosoethylurea Administered by Intraperitoneal Injection. *Cancer Res.*, **32**, 893.

MAGEE, P. N. (1969) *In vivo* Reactions of Nitroso Compounds. *Ann. N. Y. Acad. Sci.*, **163**, 717.

MAGEE, P. N. & BARNES, J. M. (1967) Carcinogenic Nitroso Compounds. *Adv. Cancer Res.*, **10**, 163.

MIRSKY, A. E. (1971) The Structure of Chromatin. *Proc. natn. Acad. Sci. U.S.A.*, **68**, 2945.

OKADA, M. & SUZUKI, E. (1972) Metabolism of Butyl(4-hydroxybutyl)-nitrosamine in rats. *Gann*, **63**, 391.

ROSENKRANZ, H. S., ROSENKRANZ, S. & SCHMIDT, R. M. (1969) Effects of Nitrosomethylurea and Nitrosomethylurethan on the Physical Properties of DNA. *Biochim. biophys. Acta*, **195**, 262.

SCHOENTAL, R. (1966) Instability of Some of the Products Formed by the Action of N-methyl-N-nitrosourethane on Cysteine *in vitro*. Role of Neighbouring Groups in Enzymatic and Carcinogenic Action. *Nature, Lond.*, **209**, 148.

SCHOENTAL, R. (1967) Methylation of Nucleic Acids by *N*($^{14}$C)-methyl-N-nitrosourethane *in vitro* and *in vivo*. *Biochem. J.*, **102**, 5-C.

SCHOENTAL, R. (1969) Lack of Correlation between the Presence of 7-methylguanine in DNA and RNA of Organs and the Localisation of Tumours after a Single Carcinogenic Dose of *N*-methyl-*N*-nitrosourethane. *Biochem. J.*, **114**, 55P.

SWANN, P. F. & MAGEE, P. N. (1968) Nitrosamine-induced Carcinogenesis. The Alkylation of Nucleic Acids of the Rat by *N*-methyl-*N*-nitrosourea, Dimethylnitrosamine, Dimethylsulphate and Methylmethanesulphonate. *Biochem. J.*, **110**, 39.

SWANN, P. F. & MAGEE, P. N. (1971) Nitrosamine-induced Carcinogenesis. The Alkylation of N-7 of Guanine of Nucleic Acids of the Rat by Diethylnitrosamine, *N*-ethyl-*N*-nitrosourea and Ethylmethanesulphonate. *Biochem. J.*, **125**, 841.